# Supplementary material for: Causal interplay between lactose intolerance and gut microbiota: a combined bidirectional Mendelian randomization and in vivo validation study
Source: Front Nutr. 2026 Jun 1;13:1803337. doi: 10.3389/fnut.2026.1803337 (PMC13265576; doi:10.3389/fnut.2026.1803337)
Supplement: Supplementary file 1 [file Data_Sheet_1.zip › supplementary materials/Forward/leave one out cross validation/ebi-a-GCST90027683.finngen_R12_E4_LACTONAS.pdf]

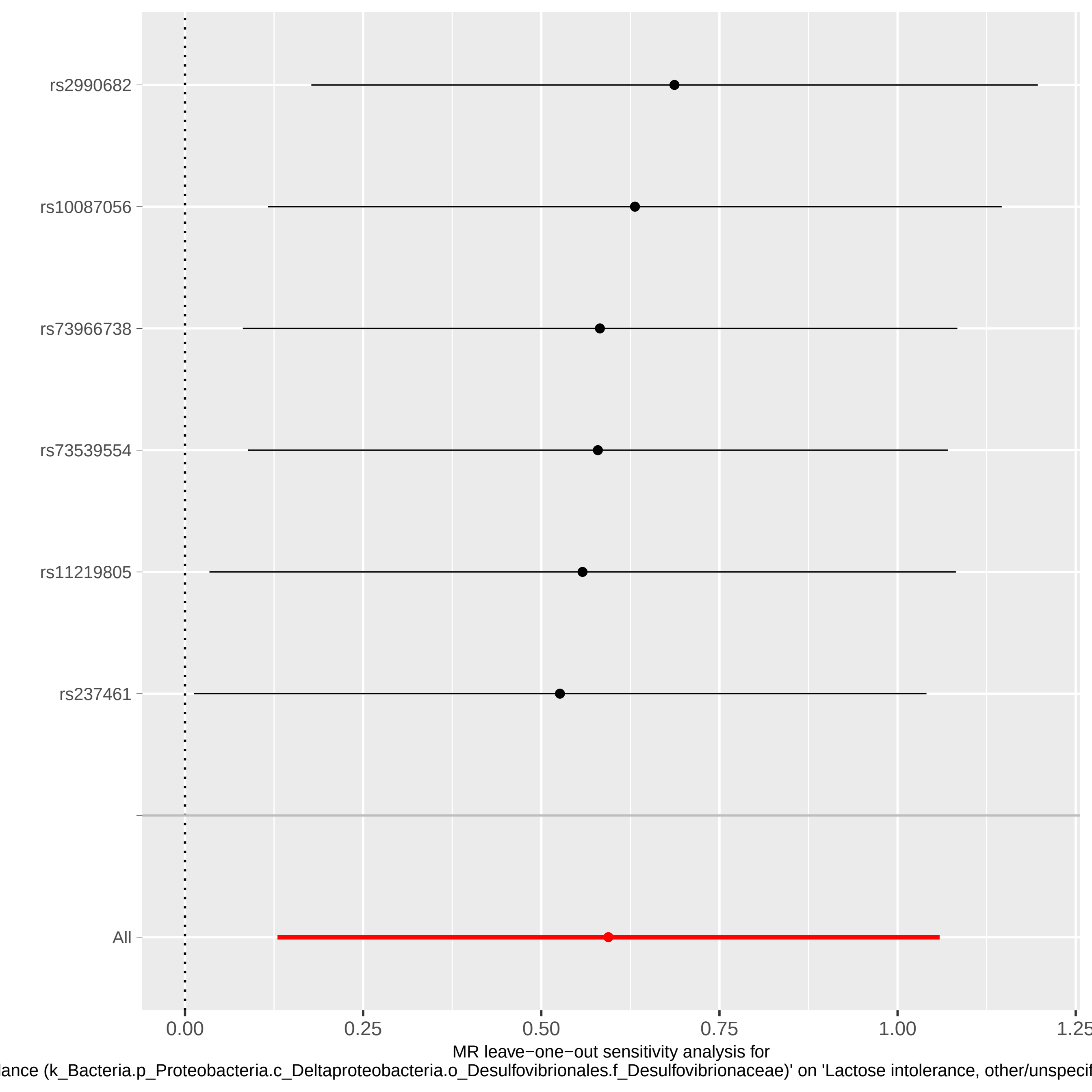
rs2990682
rs10087056
rs73966738
rs73539554
rs11219805
rs237461
All
0.00
0.25
0.50
0.75
1.00
1.25
MR leave−one−out sensitivity analysis for
lance (k_Bacteria.p_Proteobacteria.c_Deltaproteobacteria.o_Desulfovibrionales.f_Desulfovibrionaceae)' on 'Lactose intolerance, other/unspecif
